# Spontaneous Physical Activity Downregulates Pax7 in Cancer Cachexia

**DOI:** 10.1155/2016/6729268

**Published:** 2015-12-20

**Authors:** Dario Coletti, Paola Aulino, Eva Pigna, Fabio Barteri, Viviana Moresi, Daniela Annibali, Sergio Adamo, Emanuele Berardi

**Affiliations:** ^1^DAHFMO Unit of Histology and Medical Embryology, Interuniversity Institute of Myology, Sapienza University of Rome, Via Scarpa 14, 00161 Rome, Italy; ^2^Department of Biological Adaptation and Ageing B2A (CNRS UMR 8256, INSERM ERL U1164, UPMC P6), Pierre et Marie Curie University (Paris 6), 75005 Paris, France; ^3^Biology, Molecular Medicine and Nano-Biotechnologies Institute, C.N.R., Biology and Biotechnologies Department, Sapienza University of Rome, 00185 Rome, Italy; ^4^Gynaecological Oncology, Oncology Department, KU Leuven, Herestraat 49, 3000 Leuven, Belgium; ^5^Department of Kinesiology, Research Group in Exercise Physiology, KU Leuven, Tervuursevest 101, P.O. Box 1500, 3001 Leuven, Belgium

## Abstract

Emerging evidence suggests that the muscle microenvironment plays a prominent role in cancer cachexia. We recently showed that NF-kB-induced Pax7 overexpression impairs the myogenic potential of muscle precursors in cachectic mice, suggesting that lowering Pax7 expression may be beneficial in cancer cachexia. We evaluated the muscle regenerative potential after acute injury in C26 colon carcinoma tumor-bearing mice and healthy controls. Our analyses confirmed that the delayed muscle regeneration observed in muscles form tumor-bearing mice was associated with a persistent local inflammation and Pax7 overexpression. Physical activity is known to exert positive effects on cachectic muscles. However, the mechanism by which a moderate voluntary exercise ameliorates muscle wasting is not fully elucidated. To verify if physical activity affects Pax7 expression, we hosted control and C26-bearing mice in wheel-equipped cages and we found that voluntary wheel running downregulated Pax7 expression in muscles from tumor-bearing mice. As expected, downregulation of Pax7 expression was associated with a rescue of muscle mass and fiber size. Our findings shed light on the molecular basis of the beneficial effect exerted by a moderate physical exercise on muscle stem cells in cancer cachexia. Furthermore, we propose voluntary exercise as a physiological tool to counteract the overexpression of Pax7 observed in cancer cachexia.

## 1. Introduction

Cachexia is a multifactorial syndrome mostly associated with chronic illnesses and characterized by severe skeletal muscle atrophy. In cancer patients, cachexia is observed in 50–80% of advanced-stage malignancies and accounts for 20% of all cancer-related deaths [1, 2]. So far, major efforts to study the disruptive catabolic events occurring in cachectic muscle tissues focused on the molecular processes occurring within the fibers. Circulating proinflammatory cytokines and tumor-released factors are the best-established players triggering muscle wasting during cancer progression [3, 4]. Their activity alters muscle fiber amino acid metabolism, transport, and proteolysis, affecting protein synthesis and ultimately leading to cell death. These alterations in metabolic pathways, in combination with diminished regenerative capabilities, mediate the severe loss of skeletal muscle mass and function observed in cancer-related cachexia [1].

Although significant advances have been achieved in the understanding of the catabolic events occurring within cachectic muscle fibers, not much is known about muscle microenvironment, where the different muscle progenitor cells reside [5, 6]. We recently investigated the role of resident pools of stem cell in cachectic muscle [6]. Our findings showed that cachexia is linked to the overexpression of Pax7 in satellite cells and other myogenic precursors in both C26 colon carcinoma bearing mice and pancreatic cancer patients. Circulating tumor factors (e.g., proinflammatory cytokines) promote fiber damage, which is followed by the activation of both satellite cells and nonsatellite cells. Proinflammatory cytokines activate IKK*β*/NF-kB and this results in a subsequent deregulation of Pax7, which ultimately impairs myogenic cell differentiation [6]. Physiological Pax7 expression drives myogenic stem cell commitment, while its persistent expression in cancer cachexia has been shown to inhibit the differentiation of muscle stem cell [6]. Overall, the impairment of muscle regeneration, together with the increase in muscle degeneration, synergizes with an unbalanced muscle homeostasis, leaning toward a cachectic state [1, 6]. In particular, the reduced regenerative potential of skeletal muscle tissue in cancer cachexia represents a pivotal determinant of the pathological progression [6–8].

Loss of muscle function is another important feature of cancer cachexia directly impacting patient's quality of life and many studies suggest ameliorative effects of physical activity in cancer patients [3]. It has been shown that physical activity reduces fatigue [9], counteracts cancer therapy side effects, both during and after treatment [10, 11], and, in general, improves patients' quality of life [3, 12]. Specifically, systematic review of 16 randomized clinical trials for different types and stages of malignancies showed that both aerobic and resistance exercises counteract cachexia and improve muscle strength more than usual care measures in treated cancer patients [13]. The molecular mechanisms by which physical activity prevents cancer cachexia and its beneficial effects in cachectic patients have been shown to involve the anti-inflammatory properties of specific cytokines [3, 14]. Indeed, it has been reported that physical activity reduces systemic inflammation by increasing the circulating level of IL-10, a known anti-inflammatory cytokine that acts locally to counteract muscle wasting [3, 15, 16]. Furthermore, physical activity is considered a promising intervention strategy for the prevention and the treatment of cancer-related cachexia also because of its antioxidant effects. These involve an enhanced activity of antioxidant enzymes such as superoxide dismutase (SOD) and glutathione peroxidase (GPx), which counteract ROS-mediated muscle damage [3]. Moreover, exercise improves insulin sensitivity enhancing skeletal muscle metabolism [3].

Previous studies on cancer-related muscle wasting performed in C26-bearing mice showed a strong reduction of the myogenic potential of muscle stem cells in a Pax7-dependent manner [6–8].

Here we show that the impairment of muscle regeneration after local damage in cancer cachexia is associated with a prolonged inflammation and increased Pax7 expression. Furthermore, using a voluntary wheel running exercise protocol in C26-bearing mice, we demonstrate for the first time a direct involvement of aerobic exercise in the removal of the myogenic differentiation block exerted by the persisting expression of Pax7 in cachectic muscles, associated with diminished NF-kB activation.

## 2. Materials and Methods

### 2.1. Mice

Female, 7-week-old BALB/c mice were used for this study. To induce cancer-associated cachexia, a 0.5 mm^3^ solid fragment of colon carcinoma C26 was subcutaneously implanted in the back of the animals, as previously described [17]. Two different experimental protocols were adopted. For muscle regeneration analysis, muscle damage was induced by freeze-injury two weeks after tumor implantation. A steel probe precooled in dry ice was applied to* Tibialis Anterior* (TA) muscle of anesthetized animals for 10 seconds, as previously described [18]. These mice were euthanized 3, 6, 8, and 10 days after muscle damage. Mice for running experiments were housed in standard conditions with day/night cycles of 12 hours and food* ad libitum* and euthanized 19 days after tumor implantation.

All the animal studies were performed in accordance with ARRIVE guidelines and following the three Rs rule of Replacement, Reduction, and Refinement principles [19]. Animals were treated with protocols approved by the animal experimentation ethics committee of Sapienza University of Rome, Italy.

### 2.2. Voluntary Wheel Running Exercise

To analyze the effects of voluntary wheel running, we compared mice hosted in standard versus wheel-equipped cages. Cages were prepared as previously described [20]. Briefly, all wheels were supplied with a tachometer in order to record physical activity data, including total speed, total distance covered, and their daily averages. Mice in the running groups were hosted in wheel-equipped cages from the day of tumor implant until sacrifice (19 days). Mice hosted in normal cages were considered unexercised mice.

### 2.3. Dry Weight Muscle Measurement

TA,* quadriceps femoris* (QU) and* gastrocnemius* (GA) muscles were dissected, weighed (i.e., wet weight), frozen in liquid nitrogen, subjected to lyophilization in a vacuum chamber for 18–20 hours, and weighed before defrosting (i.e., dry muscle weight). This approach was used to calculate the percentage of water content of the samples after the different treatments.

### 2.4. Histology and Histochemistry

TA muscles were dissected, embedded in tissue freezing medium (Leica, Wetzlar, Germany), and frozen in liquid nitrogen-cooled isopentane. Muscle cryosections of 8 *μ*m thickness were obtained using a Leica cryostat (Leica Biosystems). For histological analysis, the sections were stained with hematoxylin and eosin (H&E, Sigma), using standard methods.

To measure NADH transferase activity, the sections were treated as previously described [17]. Esterase staining was performed as previously reported [5]. Photomicrographs were obtained using an Axioscop 2 plus system equipped with an AxioCamHRc (Zeiss, Oberkochen, Germany) at standard 1300 × 1030 pixel resolution.

### 2.5. Immunofluorescence

Transverse cryosections were fixed in 4% paraformaldehyde for 10 min at RT. After incubation with 1% bovine serum albumin (BSA) for 30 min, samples were incubated with a polyclonal anti-laminin Ab (Sigma) (1 : 100 in BSA), followed by incubation with the anti-rabbit Alexa 568 conjugated Ab (Molecular Probes, Eugene, OR) (1 : 500 in BSA). Nuclei were stained for 3 min with 0.5 *μ*g/mL Hoechst 33342 (Sigma).

### 2.6. Western Blot Analysis

Muscles were dissected, minced, and homogenized in 0.5 mL RIPA buffer (10 mM Tris-HCl pH 7.5, 10 mM EDTA, 0.5 M NaCl, 0.5% NaDoc, and 1% NP40) supplemented with Protease Inhibitor Cocktails (Roche 11697498001 and 04906837001, Germany) using a Dounce tight pestle. The homogenate was passed through a 16G needle. Proteins (100 *μ*g) were separated by SDS-PAGE and transferred electrophoretically to nitrocellulose membrane (Amersham Piscataway, NJ). Nonspecific binding was blocked in Tris-Cl Buffered Saline Solution with 0.05% Tween-20 (TBST) containing 10% nonfat dry milk (Nestlé) Blocking Buffer (BB) overnight at +4°C and then probed 1 h with primary antibody. The following specific antibodies were used:

 Anti-Pax7 1 : 50 in BB (Hybridoma supernatant, Iowa University, IO).
 Anti-MyoD 1 : 50 in TBST (Santa Cruz).
 Anti-Desmin 1 : 50 IN BB (Sigma-Aldrich, Saint Louis, MO).
 Anti-phospho-NF-kB: p65 1 : 400 in 5% BSA (Cell Signaling, 3033).
 Anti-NF-kB p65 1 : 400 in 5% BSA (Cell Signaling, 4764).
 Anti-GAPDH 1 : 10000 (Santa Cruz).

After washing in TBST, blots were incubated with anti-mouse or anti-rabbit secondary antibody HRP-conjugated (BioRad, Hercules, CA) diluted 1 : 10000 in TBST and detected by using Super Signal West Pico Chemiluminescent Substrate (Pierce, Rockford, IL).

### 2.7. Morphometric Analysis

Morphometric analysis was performed on type IIb fibers (i.e., low NADH transferase activity) as previously described [21]. For each muscle, the cross-sectional area of all the fibers in a muscular cross section was measured and the median calculated as an index of fiber size. The Scion Image Software was used to calculate the fiber cross-sectional area.

### 2.8. Statistical Analysis

All quantitative data are presented as mean or as mean ± SEM. Statistical analysis was performed using ANOVA or by Student's *t*-test, using the software available on the VassarStats web page (http://faculty.vassar.edu/lowry/VassarStats.html). A *p* value less than 0.05 was considered significant; a *p* value less than 0.01 was considered highly significant.

## 3. Results

### 3.1. Impaired Muscle Regeneration in C26 Induced Cancer Cachexia Is Associated with Prolonged Local Inflammation

**Figure 1:**
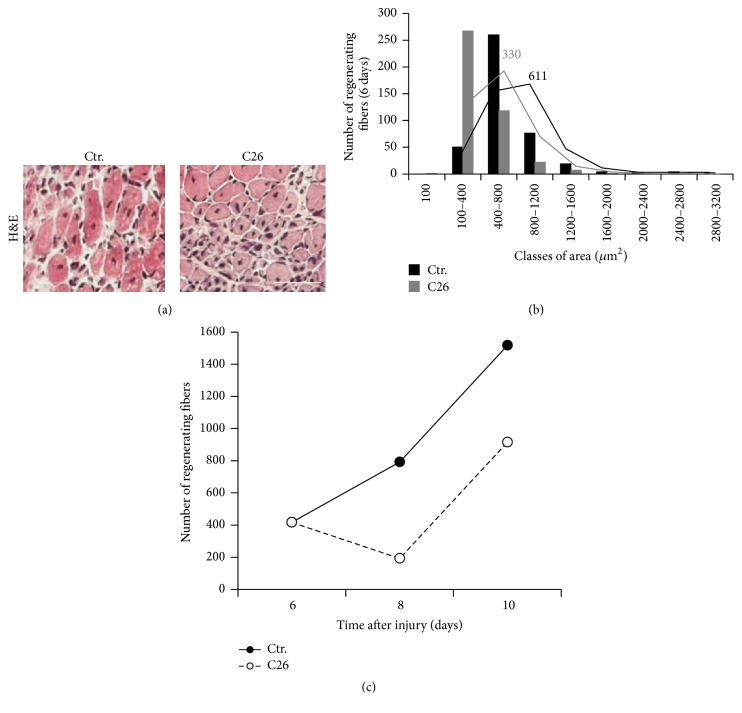
C26-bearing mice show reduced muscle regeneration after acute injury. Panel (a) depicts representative details of H&E-stained muscle cross-cryosections of the lesion sites from both control and C26-bearing mice. Bar = 100 *μ*m. (b) Morphometric analysis of regenerating fiber size from (a). Histograms show the distribution of the regenerating fiber cross-sectional area in different size classes 6 days after muscle damage (control = black bars; C26-bearing mice = gray bars). Numbers represent the median value. (c) Number of fibers with centrally located nuclei observed in the lesion site of the* tibialis* from both C26-bearing and control mice after acute damage. The total number of regenerating fibers was monitored at 6, 8, and 10 days after freeze-injury (control = black dots; C26-bearing mice = white dots).

**Figure 2:**
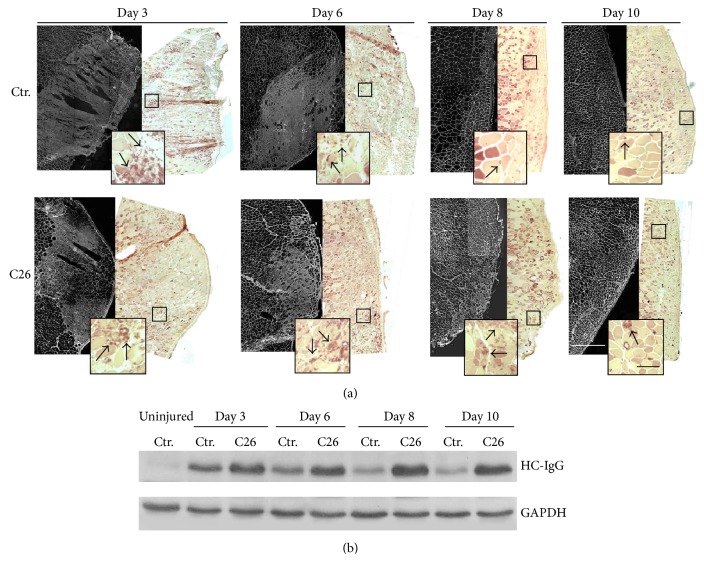
C26-bearing mice show a prolonged inflammation response during muscle regeneration. (a) Left panels: immunofluorescence analysis of IgG expression performed at the lesion site of reconstructed areas of* tibialis* muscles from both control and C26-bearing mice. (a) Right panels: esterase staining uptake at the same lesion sites highlighting the presence of macrophages in the inflamed muscles. Insects depict magnification of areas defined in the squares. Black arrows show macrophages. White bar = 0.5 mm; black bar = 100 *μ*m. (b) WB analysis of IgG expression on extracts from muscles shown in (a). First lane was loaded with the extract from a healthy, uninjured control muscle. GAPDH was used as loading control.

We analyzed muscle regeneration by histological evaluation of center-nucleated fibers 2 weeks after tumor implantation when tumors were demonstrated to begin growing exponentially and muscles developed fiber atrophy [5]. Analysis of regeneration 6 days after injury showed the presence of more mononuclear interstitial cells in the injured site of C26-bearing mice with respect to controls, indicative of a prolonged inflammatory phase following injury (Figure 1(a)). In addition, a marked deficit of regeneration was reported by the evaluation of the cross-sectional area of fibers with centrally located nuclei. At day 6 of regeneration, the mean area of regenerating fibers in muscles from C26-bearing mice was 330 *μ*m^2^, approximately half of the area of regenerating fibers in muscles derived from control mice, which was 611 *μ*m^2^(Figure 1(b)). The delay in the number of regenerating fibers was increasing over time, compared with control muscles which showed a bigger number of regenerating fibers at 8 and 10 days after damage (Figure 1(c)). Further confirmation of a hampered muscle regeneration capability in cancer cachexia came from both histological and WB analyses of IgG expression (Figures 2(a) and 2(b)). Increasing expression of IgG was observed in regenerating muscles of tumor-bearing mice at all time points analyzed following injury (with a peak at day 8), while an opposite trend in the expression profile was detected in muscles from control mice (Figures 2(a) and 2(b)). In line with these findings, histological analyses of focal lesions revealed a higher burden of recruited macrophage cells within the regenerative area of muscles from C26-bearing mice at all time points, with a peak at day 8 after muscle damage (Figure 2(a)), in agreement with the IgG expression pattern.

### 3.2. Muscle Regeneration after Acute Damage in Cachectic Muscle Is Associated with Prolonged Pax7 Expression

**Figure 3:**
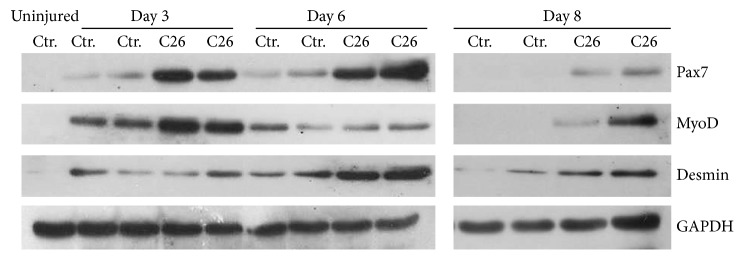
Early regenerative marker expression pattern in injured muscles. Representative WB images for Pax7, MyoD, and Desmin at 3, 6, and 8 days after freeze-injury. Blots were performed in duplicate for both control and C26-bearing mice. The first lane was loaded with the extract from a healthy, uninjured control muscle. GAPDH was used as loading control.

WB analysis of regenerative markers confirmed that C26 negatively affected muscle regeneration (Figure 3). Indeed, Pax7, MyoD, and Desmin expressions were significantly higher in muscles from C26-bearing mice than in controls, up to 8 days following injury, when these markers were almost undetectable in control muscles, a sign of completed regeneration (Figure 3). Interestingly, Pax7 expression in C26-bearing mice decreased at day 8 of regeneration, allowing MyoD to start the regenerative process, while this stage appears already completed in control muscles (Figure 3).

### 3.3. Voluntary Wheel Running Removes Pax7-Mediated Block of Myogenic Differentiation in Cancer Cachexia and Rescues Muscle Physiology

**Figure 4:**
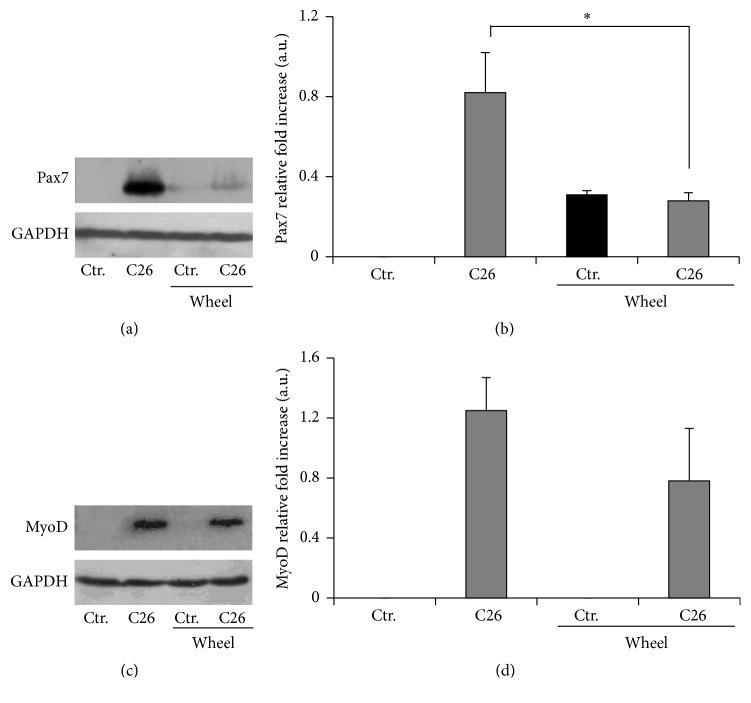
Exercise effects on myogenic markers. (a) Representative WB of Pax7 expression of muscle extracts from mice treated as indicated. (b) Densitometric analysis of Pax7 quantification. GAPDH was used as loading control. (c) Representative WB of MyoD expression of muscular extract from mice treated as indicated. (d) Densitometric analysis of MyoD quantification. Error bars are shown as means ± SEM of five independent experiments; ^*∗*^ *p* < 0.005 by one way ANOVA.

**Figure 5:**
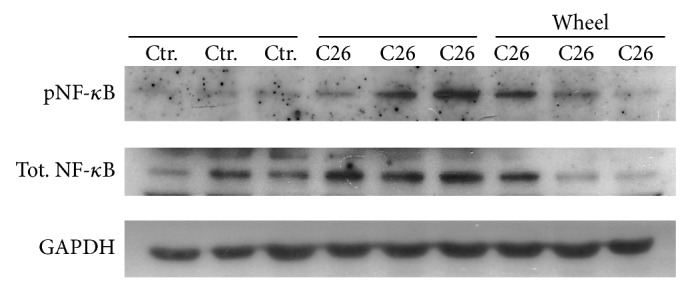
Exercise effects on NF-kB. WB of pNF-kB and total NF-kB of muscle extracts from mice treated as indicated. Each group of samples was loaded as triplicate of independent experiments. GAPDH was used as a loading control.

Cachexia is characterized by muscle damage and defective muscle regeneration associated with elevated levels of Pax7 expression [6, 7]. Genetic reduction of Pax7 expression has been shown to rescue muscle homeostasis [6]. We tested whether physical activity, known to be able to rescue muscle homeostasis in cachexia, was also sufficient to restore Pax7 physiological expression levels and promote stem cell progression from myoblast to nascent myofibers. To analyze the effects of voluntary wheel running on muscle wasting, we hosted BALB/c mice in wheel-equipped cages, as previously described [20]. Mice were divided into two groups, C26 tumor-bearing mice and healthy control mice, and individual physical activity was recorded daily by tachometers connected to the wheels. Since the exercise regimen started on the same day of tumor implantation, the approach allowed us to analyze the effects of voluntary wheel running during the development of pathological symptoms. The distance run by C26-bearing BALB/c mice was lower than that run by the healthy controls: we found nearly 50% decrease in the total as well as in the daily distance covered by cachectic mice (6 km/day versus 11 km/day, resp.) [20]. However, no significant differences were observed in average speed between cachectic and control mice, suggesting that the two groups exercised at similar intensity levels. With the aim to investigate the molecular signature of muscle response to cachexia and how it is modulated by exercise, we performed WB analysis for markers of muscle regeneration, such as MyoD and Pax7 in C26-bearing and control mice, with or without exercise. A striking upregulation of both Pax7 expression and MyoD expression in cachectic muscle was found (Figures 4(a)–4(d)). This confirmed the occurrence of myoblast activation in cancer-mediated muscle wasting, as previously reported [6]. However, we found, for the first time, that voluntary physical activity* per se* did not induce a significant increase in the expression of Pax7 (Figures 4(a) and 4(b)) and MyoD (Figures 4(c) and 4(d)), albeit it specifically downregulated Pax7 expression in the musculature of tumor-bearing mice, likely releasing a block to muscle regeneration (Figures 4(a) and 4(b)). In addition, analysis of NF-kB confirmed its increment in muscles from C26-bearing mice, as reported by other authors [22, 23]. However, enhanced voluntary physical activity, together withPax7 decrease, is also associated with a downregulation of both total and activated (p65) levels of NF-kB compared to those observed in muscles from C26-bearing mice at rest (Figure 5).

**Figure 6:**
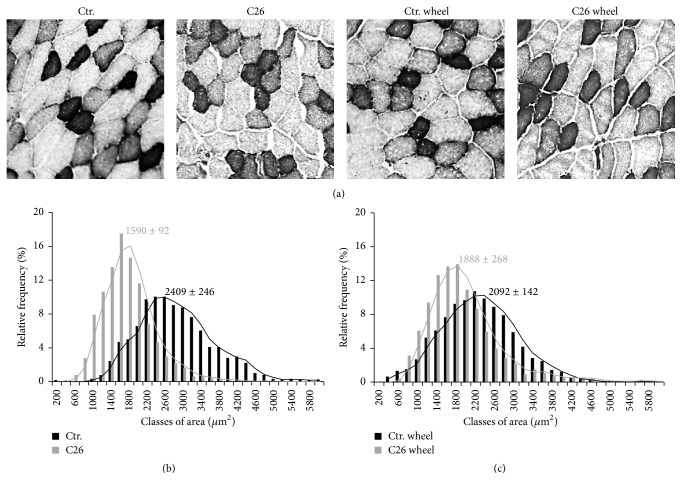
Voluntary wheel running rescues skeletal muscle atrophy in C26-bearing mice. (a) NADH staining in TA muscles. Glycolytic fibers are shown as pale colored while oxidative fibers stain as dark. Bar = 100 *μ*m. Morphometric analysis of glycolytic fibers among healthy controls (black bars) and C26-bearing mice (gray bars) at rest (b) and in the presence of voluntary wheel running (c). Numbers represent the median value ± SEM of three independent experiments (C26 versus C26 wheel: *F* = 1126; *p* = 0.0001).

Given the observed exercise-induced Pax7 downregulation to physiological levels in cachexia, we analyzed the effects of exercise on muscle homeostasis. In order to avoid the bias of a possible contribution of inflammatory edema to muscle mass, we evaluated the dry weight of TA, QU, and GA muscles in all experimental conditions to assess muscle mass. Muscles from tumor-bearing mice placed in standard cages (without wheel) showed a significant weight loss (Table 1), while wheel running was able to revert the loss of muscle mass in C26-bearing mice to levels comparable to those observed in not exercised healthy control mice (Table 1). In addition, we evaluated glycolytic fibers areas, which are highly reduced in cancer-related muscle atrophy [24] (Figures 6(a) and 6(b)) but not after exercise-induced Pax7 downregulation (Figures 6(a) and 6(c)). Overall, morphometric analyses of TA muscles from healthy controls and C26-bearing mice, with or without wheel running, showed that the latter improves muscle mass in cachectic mice by increasing fiber cross-sectional area of glycolytic fibers (C26 versus C26 wheel: *F* = 1126; *p* = 0.0001) (Figures 6(b) and 6(c)).

## 4. Discussion

Deregulated levels of Pax7 have been recently shown to contribute to muscle wasting in cancer cachexia [6, 7]. To further investigate the procachectic role of Pax7 in skeletal muscle tissue in tumor-bearing mice, we characterized the cellular response to muscle damage occurring in muscles obtained from cachectic mice after acute damage. In a previous work, we exploited a pharmacologically or genetically induced Pax7 downregulation to obtain rescue of muscle homeostasis [6]. In this study, we demonstrate that the same result/effect can be obtained by a physiological regulation of Pax7 expression mediated by physical activity. The latter may have readily applicable translational implications.

Upon focal injury of both healthy and C26-bearing mice, histological evaluations showed a considerable delay of muscle regeneration in C26-bearing mice compared with healthy controls, both in terms of quantity (number) and quality (size) of the regenerating fibers involved. Furthermore, the early phases following the mechanical destruction of both fibers and connective tissue were characterized by a significant accumulation of infiltrating mononuclear cells. This massive recruitment of inflammatory cells is confirmed by high immunoglobulins expression, which appears stronger and prolonged over time in muscle from C26-bearing mice compared to controls.

Our previous study on the C26 cachectic model showed that, overall, neutrophils and lymphocytes do not accumulate within the stromal compartment of cachectic muscles, whereas the macrophage content declines [5]. Interestingly, esterase staining reveals that macrophages are the most abundant interstitial mononuclear cells in muscles from C26-bearing mice after acute damage. We noticed a prolonged expression of Pax7 in muscles from C26-bearing mice after acute injury. Likely, this elevated Pax7 expression represents an inhibitory signal keeping satellite cells in an undifferentiated, proliferating status. Since macrophages promote fiber membrane repair during regeneration [25], while the overexpression of Pax7 triggers the onset of muscle wasting [6], the asynchronous trend observed between weaves of events involved in divergent homeostatic processes mirrors the remarkable delay in muscle regeneration observed in cachectic mice. Pax7 deregulation also impacts the functionality of MRFs [6, 26, 27]. We found that voluntary wheel running, considered as a low-intensity [28, 29] and aerobic model of exercise [3], downregulates Pax7 closely to the control levels, thus reestablishing the typical expression pattern observed in healthy muscle. Moreover, the absence of MyoD and the early detectable presence of Pax7 in muscles from exercised healthy controls confirm that voluntary free running is associated with skeletal muscle adaptations related to an increased anabolism [3, 30].

Within the molecular adaptation of skeletal muscles mediated by physical activity in cancer cachexia, we also observed a downregulation of total and activated NF-kB levels in C26-bearing mice hosted in wheel-equipped cages compared to those hosted in standard cages. These data confirmed the pivotal role of NF-kB observed in cancer-related muscle wasting [22, 23] and showed that the decline of Pax7 during voluntary wheel running is associated with a reduction of NF-kB activity, further suggesting a regulatory link between the two factors, as previously demonstrated [6].

In summary, here we demonstrate that, in addition to the already known beneficial effects in cancer patients [29–31], physical activity downregulates Pax7 and restores muscle mass by increasing glycolytic fiber size. Indeed, muscle weight analysis revealed a significant rescuing effect of exercise against muscle weight loss. Several clinical reports showed an abnormal high level of Pax7 in skeletal muscle tissue from patients with different cancers, including gastric [32] and pancreatic tumors [6] and rhabdomyosarcomas [33]. Thus, the possibility to counteract Pax7 overexpression by adopting a protocol of low-intensity physical exercise, compatible with the clinical features of the primary disease, may represent an important tool to be used in association with the common therapeutic strategies.

## 5. Conclusion

Pax7 is one of the key mediators of the impaired myogenic ability observed in cancer-induced cachexia. Our data demonstrate that in cachectic mice, displaying a prolonged Pax7 expression, muscle regeneration after an acute damage is delayed, compared to controls. In addition, we showed that in C26 tumor-bearing mice voluntary wheel running downregulates Pax7 expression to levels similar to those observed in not exercised healthy mice.

Taken together, our findings suggest that the beneficial effects of a moderate physical activity on cachectic muscles are mediated, at least in part, by its ability to downregulate the expression of Pax7 and the activation of NF-kB, thus removing the myogenic differentiation block observed in cancer cachexia.

## Figures and Tables

**Table 1:** Muscle dry weight analyses.

Muscles	Treatments	Muscle dry weight (mg)	
		Not exercised	Exercised
Tibialis	Ctr.	11.32 ± 0.5	12 ± 0.6
	C26	9.68 ± 0.5^*∗*^	11 ± 0.6

Gastrocnemius	Ctr.	23.4 ± 0.1	26.7 ± 1.4
	C26	19.3 ± 1.5^*∗*^	20.7 ± 1.5

Quadriceps	Ctr.	15.6 ± 0.6	14.8 ± 0.7
	C26	9.4 ± 0.4^*∗*^	11.9 ± 0.2^*∗*^

Dry weight analyses of tibialis (TA), gastrocnemius (GA), and quadriceps (QU) muscles from control and C26-bearing mice in the presence or absence of 19 days of voluntary free running. (*n* = 5; ^*∗*^ *p* < 0.05 versus Ctr. by Student's *t*-test).
